# Impact of Primary Health Care Data Quality on Infectious Disease Surveillance in Brazil: Case Study

**DOI:** 10.2196/67050

**Published:** 2025-02-21

**Authors:** Pilar Tavares Veras Florentino, Juracy Bertoldo Junior, George Caique Gouveia Barbosa, Thiago Cerqueira-Silva, Vinicius de Araújo Oliveira, Marcio Henrique de Oliveira Garcia, Gerson Oliveira Penna, Viviane Boaventura, Pablo Ivan Pereira Ramos, Manoel Barral-Netto, Izabel Marcilio

**Affiliations:** 1 Centro de Integração de Dados e Conhecimento em Saúde (CIDACS), Instituto Gonçalo Moniz, Fundação Oswaldo Cruz, R. Mundo, 121 - sala 315 - Trobogy, Salvador, 41745-715, Brazil, 55 7131762357; 2 Department of Medical Statistics, London School of Hygiene & Tropical Medicine, London, United Kingdom; 3 Secretaria de Atenção Primária, Ministério da Saúde, Brasília, Brazil; 4 Secretaria de Vigilância em Saúde e Ambiente, Ministério da Saúde, Brasília, Brazil; 5 Escola Fiocruz de Governo, Fundação Oswaldo Cruz (Fiocruz), Rio de Janeiro, Brazil; 6 Núcleo de Medicina Tropical, Universidade de Brasília, Brasília, Brazil; 7 Laboratório de Medicina e Saúde Pública de Precisão, Instituto Gonçalo Moniz, Fundação Oswaldo Cruz, Salvador, Brazil; 8 Departamento de Epidemiologia, Escola Bahiana de Medicina e Saúde Pública, Salvador, Brazil

**Keywords:** primary health care, data quality, infectious disease surveillance, Brazil, early warning system

## Abstract

**Background:**

The increase in emerging and re-emerging infectious disease outbreaks underscores the need for robust early warning systems (EWSs) to guide mitigation and response measures. Administrative health care databases provide valuable epidemiological insights without imposing additional burdens on health services. However, these datasets are primarily collected for operational use, making data quality assessment essential to ensure an accurate interpretation of epidemiological analysis. This study focuses on the development and implementation of a data quality index (DQI) for surveillance integrated into an EWS for influenza-like illness (ILI) outbreaks using Brazil’s a nationwide Primary Health Care (PHC) dataset.

**Objective:**

We aimed to evaluate the impact of data completeness and timeliness on the performance of an EWS for ILI outbreaks and establish optimal thresholds for a suitable DQI, thereby improving the accuracy of outbreak detection and supporting public health surveillance.

**Methods:**

A composite DQI was established to measure the completeness and timeliness of PHC data from the Brazilian National Information System on Primary Health Care. Completeness was defined as the proportion of weeks within an 8-week rolling window with any register of encounters. Timeliness was calculated as the interval between the date of encounter and its corresponding registry in the information system. The backfilled PHC dataset served as the gold standard to evaluate the impact of varying data quality levels from the weekly updated real-time PHC dataset on the EWS for ILI outbreaks across 5570 Brazilian municipalities from October 10, 2023, to March 10, 2024.

**Results:**

During the study period, the backfilled dataset recorded 198,335,762 ILI-related encounters, averaging 8,623,294 encounters per week. The EWS detected a median of 4 (IQR 2‐5) ILI outbreak warnings per municipality using the backfilled dataset. Using the real-time dataset, 12,538 (65%) warnings were concordant with the backfilled dataset. Our analysis revealed that 100% completeness yielded 76.7% concordant warnings, while 80% timeliness resulted in at least 50% concordant warnings. These thresholds were considered optimal for a suitable DQI. Restricting the analysis to municipalities with a suitable DQI increased concordant warnings to 80.4%. A median of 71% (IQR 54%-71.9%) of municipalities met the suitable DQI threshold weekly. Municipalities with ≥60% of weeks achieving a suitable DQI demonstrated the highest concordance between backfilled and real-time datasets, with those achieving ≥80% of weeks showing 82.3% concordance.

**Conclusions:**

Our findings highlight the critical role of data quality in improving the EWS’ performance based on PHC data for detecting ILI outbreaks. The proposed framework for real-time DQI monitoring is a practical approach and can be adapted to other surveillance systems, providing insights for similar implementations. We demonstrate that optimal completeness and timeliness of data significantly impact the EWS’ ability to detect ILI outbreaks. Continuous monitoring and improvement of data quality should remain a priority to strengthen the reliability and effectiveness of surveillance systems.

## Introduction

In recent decades, the world has witnessed an unprecedented surge of emerging and re-emerging infectious disease outbreaks, underscoring the need for stronger early warning systems (EWSs) [1,2]. The widespread and growing use of electronic health records (EHRs) has heightened the demand for automated processes in disease surveillance [3,4].

Systematic monitoring of administrative health care databases provides valuable epidemiological insights [5,6]. Importantly, using administrative data for health surveillance avoids the overburden of surveillance teams while ensuring timeliness, as no duplication of registry is required [3]. This cost-efficient approach enhances the ability to detect outbreaks, particularly in low-resource settings, thus contributing to global security [3].

However, an effective automated EWS based on administrative datasets requires that a real-time data quality assessment algorithm is set within the EWS pipeline. Since administrative data are primarily collected for operational purposes, assessing their quality is crucial to accurate interpretation of epidemiological analysis [7,8]. A systematic review on EHR data quality assessment studies found 14 articles describing dedicated data quality programs deployed in real-world settings, while only 4 produced results generally applicable in diverse settings. Ozonze et al [9] suggest there is an absence of comprehensive tools for facilitating reliable and consistent data quality assessments.

Moreover, despite existing methods for evaluating the quality of administrative health data, including EHR data quality assessment [8] and indicators for specific programs such as the Data Quality Audit and the Data Quality Self-Assessment for immunization data [10], there remains a gap in applying similar methods to data used for health surveillance. Although metrics for assessing the quality of surveillance systems are well established [3], to the best of our knowledge, these have not been applied to evaluate administrative data when used for epidemiological surveillance purposes.

This paper describes the development and implementation of a data quality index (DQI) to assess the quality of administrative data used in epidemiological surveillance systems. We focus on applying the DQI to nationwide Brazilian primary health care (PHC) administrative data integrated into an EWS for influenza-like illness (ILI) outbreaks. The study compares the EWS performance across different DQI levels, addressing a critical gap in current research by establishing metrics that ensure accurate and timely outbreak detection while leveraging the cost-efficiency of administrative health databases.

## Methods

### Study Design

We developed and implemented a data quality assessment algorithm within ÆSOP (Alert-Early System of Outbreaks with Pandemic Potential), a previously validated EWS [11]. This EWS applies aberration detection algorithms, such as the Early Aberration Reporting System (C2) [12], to a time series consisting of weekly counts of ILI-related PHC encounters per municipality, aiming at the early detection of outbreaks. To assess the data quality of the PHC data stream, we established the composite indicator DQI to measure the completeness and timeliness of the data. Using the backfilled PHC dataset as a gold standard, we evaluated the impact of data quality in the EWS’ performance using different levels of data quality of the weekly updated real-time PHC dataset across all 5570 Brazilian municipalities from October 10, 2023, to March 10, 2024.

### Data Source

Brazil is an upper middle-income country with approximately 212.6 million people living in 5570 municipalities [13], and we included all ILI-related PHC encounters occurring during the study period in our analysis. We analyzed data from the Brazilian Unified Health System (SUS), which stands as one of the largest public health systems globally, providing comprehensive and universal health care to the entire population. The effective management of SUS relies on diverse information systems, among which the Brazilian National Information System on Primary Health Care (SISAB [Sistema de Informação em Saúde para a Atenção Básica]) plays a crucial role. SISAB is a hierarchical, decentralized information system maintained and managed by the Ministry of Health (MoH), and harbors data on all publicly funded PHC encounters in the country. Data registration is mandatory for the allocation of financial resources from the federal to the municipal level. All encounters are coded by the *ICD-10* (*International Statistical Classification of Diseases, Tenth Revision*) or the International Classification of Primary Care (ICPC-2).

According to the MoH’s guidelines, municipalities are requested to update the system at least on a monthly basis, with a window of 4 months for amendments following each monthly submission. This operational guideline aligns with the SISAB’s purpose of informing decision-making for the management of the PHC system in the country. However, the EWS uses weekly updates of the SISAB database to detect ILI outbreaks. Therefore, this real-time, weekly updated dataset may present incompleteness and a temporal lag between the dates of encounter and data registration into the system (Figure S1 in Multimedia Appendix 1).

### The DQI

We defined the dimensions of completeness and timeliness to develop quantitative indicators for monitoring data quality in the EWS. Completeness is one of the most commonly used dimensions in data quality assessment and may be defined as the proportion of data filled with values for each attribute or entity in the database, while timeliness can be defined as the availability of data for decision-making, measured by the time interval between the occurrence of the measured event and its capture in an information system [14].

In our study, completeness refers to the proportion of weeks in each 8-week rolling window with any register of a PHC encounter. The indicator is measured as a fraction, with the numerator ranging from 0 to 8, and the denominator is 8 weeks, which is expressed as a percentage. Timeliness refers to the time interval, in number of weeks, between the date of the PHC encounter and its registry in the database. The indicator is represented by the proportion of registries occurring in 2 weeks or less from the PHC encounter in the same 8-week rolling window.

As it is recommended that the diverse quality dimensions should be collectively analyzed for a more comprehensive evaluation of data quality [15], we combined the 2 selected indicators in a composite measure, named DQI. The DQI is assessed weekly, for each municipality, once the PHC data are updated into the EWS pipeline.

### Impact of DQI on the EWS’ Performance

To decide on the minimum required threshold of completeness and timeliness to derive trustworthy results with the EWS, we applied the EWS algorithm to the retrospectively gathered, backfilled PHC dataset. We compared the results to those obtained when applying the EWS to the weekly updated, herein named real-time PHC dataset (Figure S1 in Multimedia Appendix 1). Using the backfilled dataset as a reference, we calculated the proportion of concordant warnings detected in the real-time dataset. Accordingly, the DQI is expressed as either “suitable” or “unsuitable” when the minimum threshold of both completeness and timeliness is reached, indicating that the data quality may not be adequate for reliable EWS outputs.

Analyses were performed using Python (version 3.9) and R (version 4.3.1) software. The database’s description and the scripts are available on GitHub [16].

### Ethical Considerations

The study protocol and procedures were reviewed and approved by the Ethical Review Board of Oswaldo Cruz Foundation – Fiocruz Bahia (protocol CAAE 61444122.0.0000.0040).

Data on publicly funded PHC encounters were collected and compiled by the MoH for funding reasons. No consent was needed for data collection at this administrative level. For this study, we accessed an aggregated database consisting of the number of encounters per epidemiological week, per municipality, and per diagnostic code. The accessed database has no information at the individual level, and given that this study involves secondary analysis of existing deidentified data and does not involve direct interaction with human participants, it is classified as exempt from the requirement for informed consent under applicable ethical guidelines.

## Results

There were 198,335,762 recorded ILI-related encounters in the backfilled PHC dataset, which corresponds to an average of 8,623,294 encounters per week between October 10, 2023, and March 10, 2024. Using the backfilled dataset, the EWS detected a median of 4 (IQR 2‐5) warnings of ILI outbreaks per municipality in the study period.

**Figure 1.**
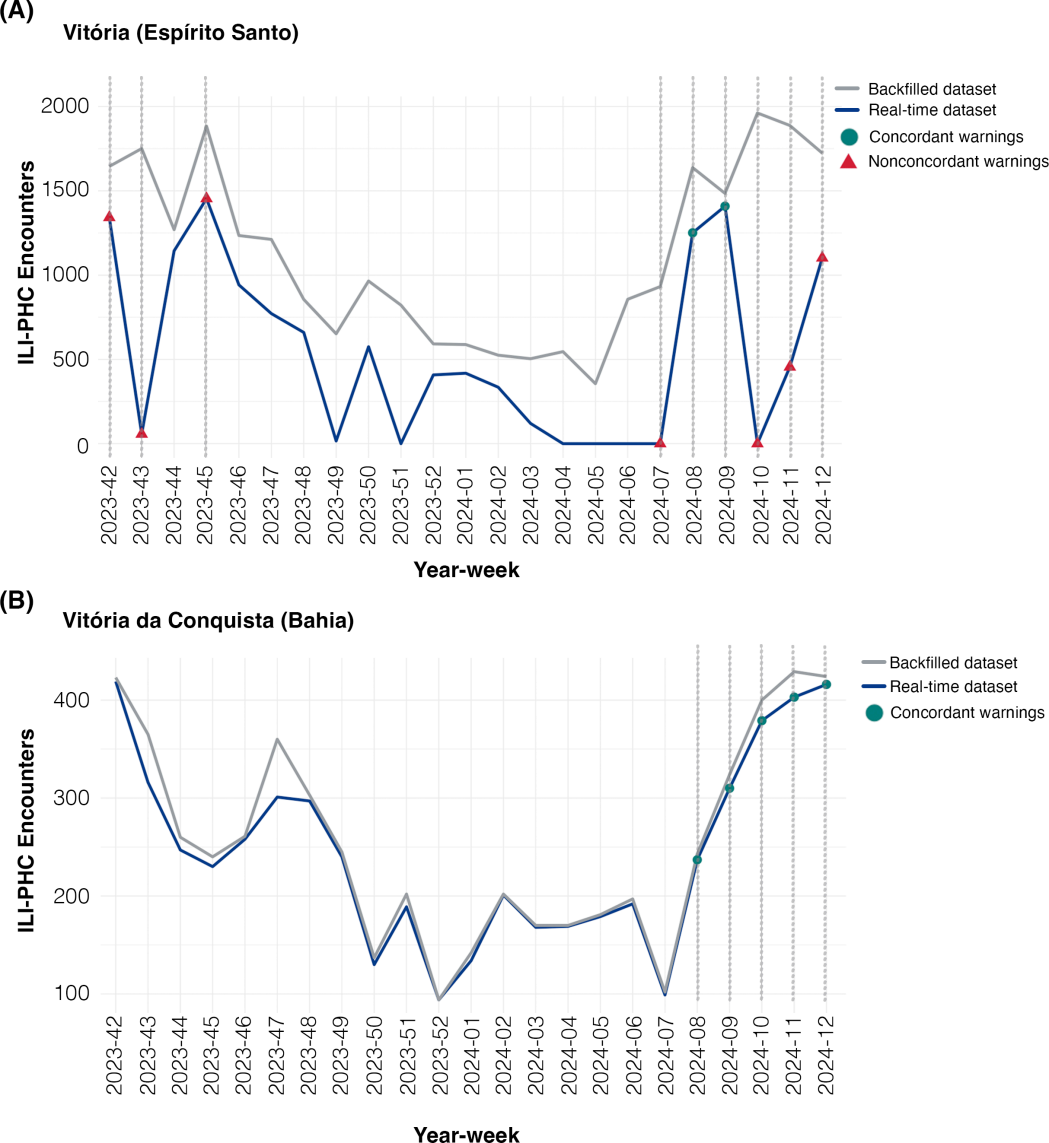
Primary Health Care encounters due to influenza-like illness per week in (A) Sao Paulo (municipality with a suitable data quality index in less than 60% of the 23 weeks in study period) and (B) Vitoria da Conquista (municipality with a suitable data quality index for over 80% of the study period). Plots show backfilled (gray line) and real-time (green line) PHC datasets for influenza-like illness (ILI) encounters. Vertical dashed lines show all detected warnings with the Early Aberration Reporting System (EARS), red triangles show nonconcordant warnings between backfield and real-time datasets, and blue circles show concordant warnings between them. ILI: influenza-like illness; PHC: Primary Health Care.

**Figure 2.**
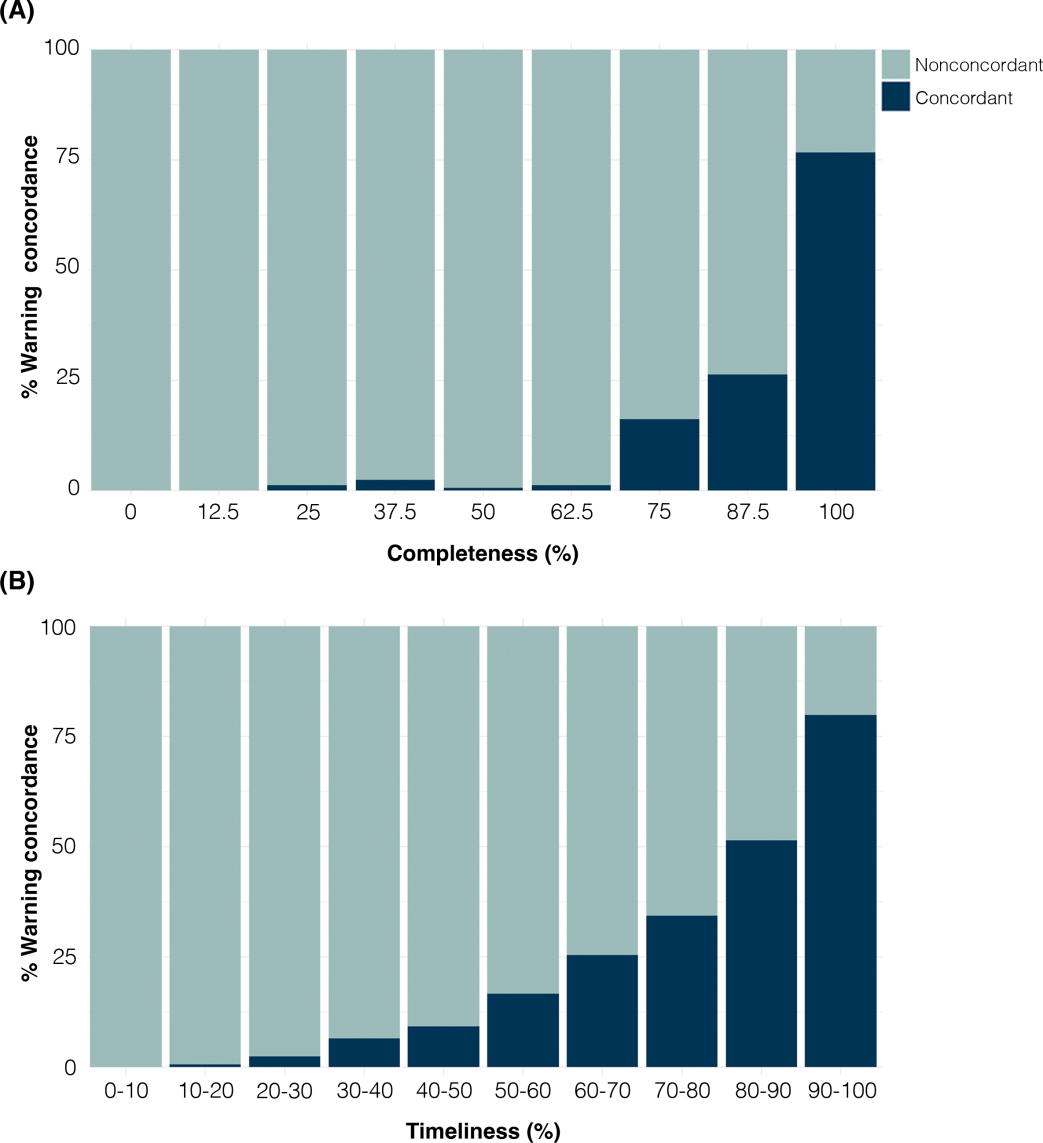
Proportion of concordant outbreak warnings detected in backfilled and real-time Brazilian Primary Health Care datasets. Outbreak warnings generated by the Early Aberration Reporting System (EARS) were identified in both backfilled and real-time datasets. Concordant warnings, detected in both datasets within the same week, are represented in dark blue, while nonconcordant warnings, identified only in the backfilled dataset, are shown in light blue. The analysis considers the proportion of concordant and nonconcordant warnings based on real-time dataset. (**A**) Completeness: the percentage of records from the real-time dataset in each 8-week rolling window (ranging from 0% to 100%) and (**B**) timeliness: the proportion of records registered within 2 weeks or less of the PHC encounter, measured within the same 8-week rolling window (ranging from 0% to 100%).

**Figure 3.**
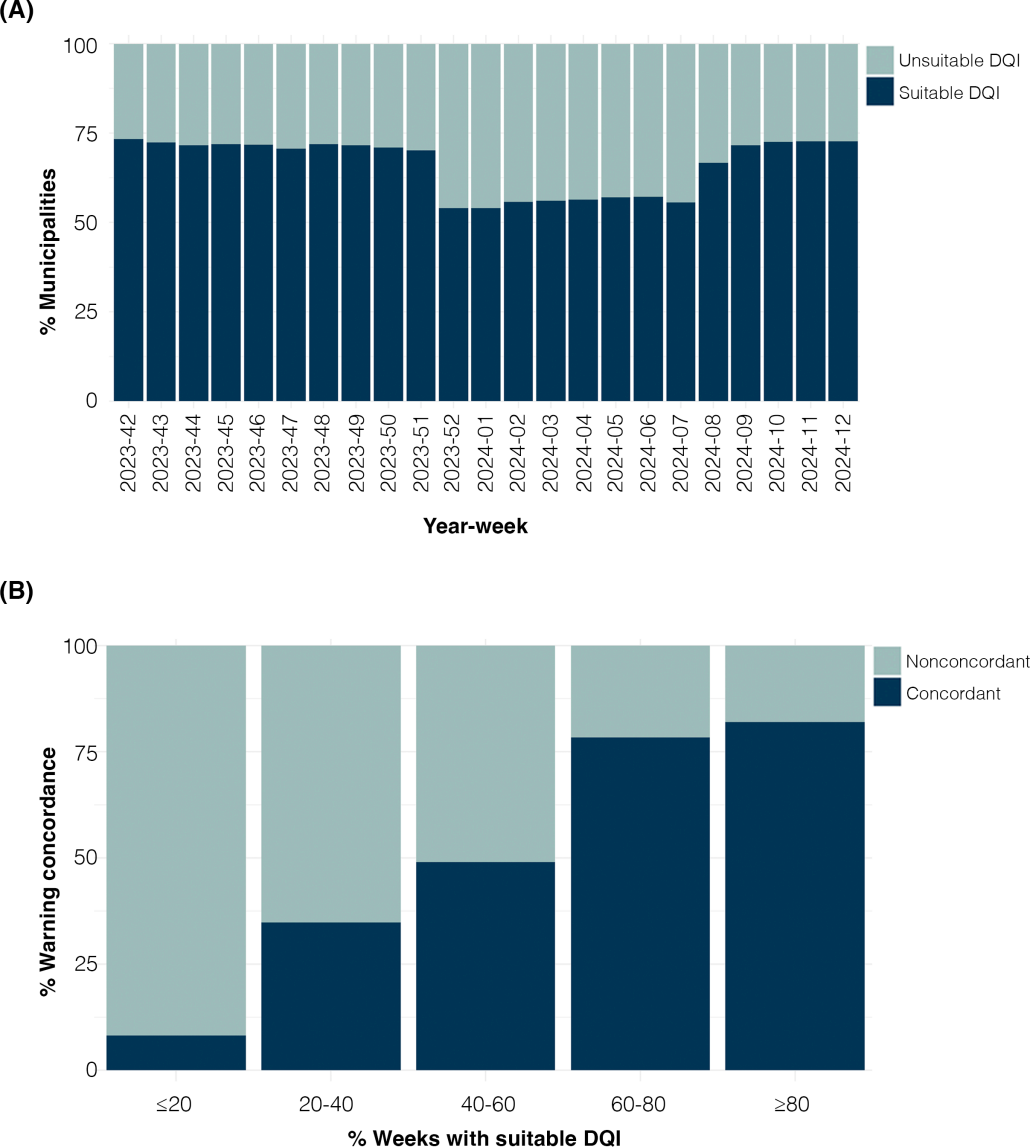
Proportion of Brazilian municipalities with suitable data quality index (DQI) and concordant warnings over time. (**A**) Weekly analysis of the proportion of municipalities with a suitable DQI from epidemiological week 42 of 2023 to week 12 of 2024. (**B**) Proportion of concordant warnings (dark blue), identified in both datasets within the same week, and nonconcordant warnings (light blue), detected only in the backfilled dataset. The analysis is based on the proportion of weeks with a suitable DQI in the real-time dataset from Brazilian municipalities.

Figure 1 illustrates the impact of the DQI on the ability of the EWS to correctly identify potential ILI outbreaks. Using the real-time dataset, the EWS detected 12,538 (65%) warnings of ILI-outbreaks that were concordant with warnings detected in the backfilled dataset (Table S1 in Multimedia Appendix 1). The proportion of concordant warnings detected in the real-time dataset, based on different levels of completeness (Figure 2A and Table S1 in Multimedia Appendix 1) and timeliness (Figure 2B and Table S1 in Multimedia Appendix 1), indicated that 100% completeness and a minimum of 80% timeliness yielded the highest percentage of concordant warnings. Therefore, these values were established as the thresholds for grading the DQI as suitable or unsuitable for the EWS. Restricting the EWS analysis to municipalities with a suitable DQI, the proportion of warnings for ILI outbreaks concordant to the backfilled dataset increased to 80.4% (Table S2 in Multimedia Appendix 1). We found a median of 71% (IQR 54%‐71.9%) of Brazilian municipalities with a suitable DQI per week in the study period (Figure 3A and Table S2 in Multimedia Appendix 1).

Additionally, we analyzed concordant warnings by grouping municipalities based on the proportion of weeks in which they exhibited a suitable DQI (≤20%, 20%‐40%, 40%‐60%, 60%‐80%, and ≥80%). Our findings revealed that municipalities with over 60% of weeks featuring a suitable DQI had the highest proportion of concordant warnings between the backfilled and real-time datasets (Figure 3B, Table S2 in Multimedia Appendix 1).

## Discussion

### Principal Findings

Our study highlights the critical role of data quality in the performance of the EWS for infectious disease surveillance using PHC data. In addition, we provide a practical approach for monitoring data quality in real time, which can be adapted to other settings and data types. Our findings revealed that municipalities with over 60% of weeks featuring a suitable DQI had the highest proportion of concordant warnings between the backfilled and real-time datasets. Introducing the DQI as an algorithm integrated into the EWS can guide data management practices and inform decision-making processes.

Similar to our findings, a recent systematic review of the effectiveness of EWS found that the improvement of data is pivotal for emergency department–based surveillance [17]. However, efforts for automatization of data quality assessment are typically scattered [9], and the literature on the operationalization of data quality assessment remains scarce. A study on data quality assessment for public health information systems found a lack of systematic procedures for quality assessment. While quality assessment of quantitative data generally used descriptive surveys, the authors argued about the importance of systematic scientific data quality assessment [18]. To the best of our knowledge, this is the first publication to assess the importance of integrating data quality monitoring into an EWS.

Fulcher et al [19] demonstrated how administrative health data were successfully used to implement a syndromic surveillance system during the COVID-19 pandemic. However, the process of cleaning data and handling missed data was carried out by a dedicated analyst once the updated database became available [19]. We anticipate that the framework for a data quality assessment integrated to the EWS pipeline presented here can be adapted to other surveillance systems and can provide insights for similar implementations.

Using a retrospectively gathered, backfilled PHC dataset, we evaluated the EWS based on optimal data quality conditions. However, administrative data usually exhibit incompleteness and delays, and the EWS should be capable of detecting outbreaks using the available dataset in real time. Our analysis revealed that high levels of completeness (100%) and timeliness (at least 80%) are necessary to achieve the highest proportion of concordant warnings between backfilled and real-time datasets. Additionally, our results indicate that even incremental data quality improvements substantially enhance the EWS’ performance. Achieving such high standards may pose challenges, particularly in low-resource settings that potentially face limitations due to infrastructure such as unreliable internet connectivity and insufficient computer power. Despite these challenges, we found a weekly median of 71% of Brazilian municipalities achieving the threshold for a suitable DQI for the EWS. This result suggests that a significant proportion of municipalities met the minimum threshold for data quality even in constrained settings.

In this study, we used the SUS database, which covers approximately 75% of the Brazilian population, with great granularity, reaching underserved rural and remote regions [20]. This approach allowed us to assess the performance of the EWS across different regions and health service contexts. However, these findings may not be directly applicable to other countries. It is likely that the use of the EWS in different health system structures and data management practices will need adjustments and may require distinct data quality requirements [8].

Another limitation of this study is that we could not access other dimensions of data quality. Specifically, we could not access the accuracy of registers in the PHC dataset. Accuracy represents the extent to which the data are free of error and reliable [8,9]. We worked with aggregated, secondary data, and did not have access to the complete EHRs, which precluded us from verifying whether the diagnostic codes in the database accurately reflected patients’ main clinical problems. It is our perspective that evaluating the accuracy of the *ICD-10* and ICPC-2 is of great importance. However, given the large numbers of PHC encounters registered weekly, misclassifications of the reason of encounter are likely to be nondifferential. Additionally, syndromic surveillance systems are designed to operate effectively even with some level of imprecision, as their primary purpose is to detect patterns and trends rather than to provide definitive diagnoses.

### Conclusion

Our findings demonstrate that implementing a robust and integrated DQI analysis can significantly enhance the EWS’ ability to detect ILI outbreaks, contributing to better public health outcomes and ultimately to global health security. Beyond contributing to the existing literature on EWS, this study highlights the importance of systematic data quality assessment. Continuous monitoring and improvement of data quality should be prioritized to ensure the reliability and effectiveness of surveillance systems. Additionally, our study suggests that similar frameworks can be adapted to different contexts. As health systems increasingly use digital health data for decision-making, our approach represents a model for integrating data quality monitoring into surveillance systems, ultimately enhancing the capacity to detect and respond to infectious disease outbreaks effectively.

## Supplementary material

10.2196/67050 Multimedia Appendix 1 Additional material.

10.2196/67050 Multimedia Appendix 2 Conversation with the chatbot for grammatical revision.

## Abbreviations

DQI: data quality index
EHR: electronic health record
EWS: early warning system
*ICD-10*: *International Statistical Classification of Diseases, Tenth Revision*
ICPC-2: International Classification of Primary Care
ILI: influenza-like illness
MoH: Ministry of Health
PHC: Primary Health Care
SISAB: Sistema de Informação em Saúde para a Atenção Básica
SUS: Brazilian Unified Health System
ÆSOP: Alert-Early System of Outbreaks with Pandemic Potential
