# Supplementary material for: Impact of Primary Health Care Data Quality on Infectious Disease Surveillance in Brazil: Case Study
Source: JMIR Public Health Surveill. 2025 Feb 21;11:e67050. doi: 10.2196/67050 (PMC11870279; doi:10.2196/67050)
Supplement: Multimedia Appendix 1 [file publichealth-v11-e67050-s001.docx]

**MULTIMEDIA APPENDIX 1**


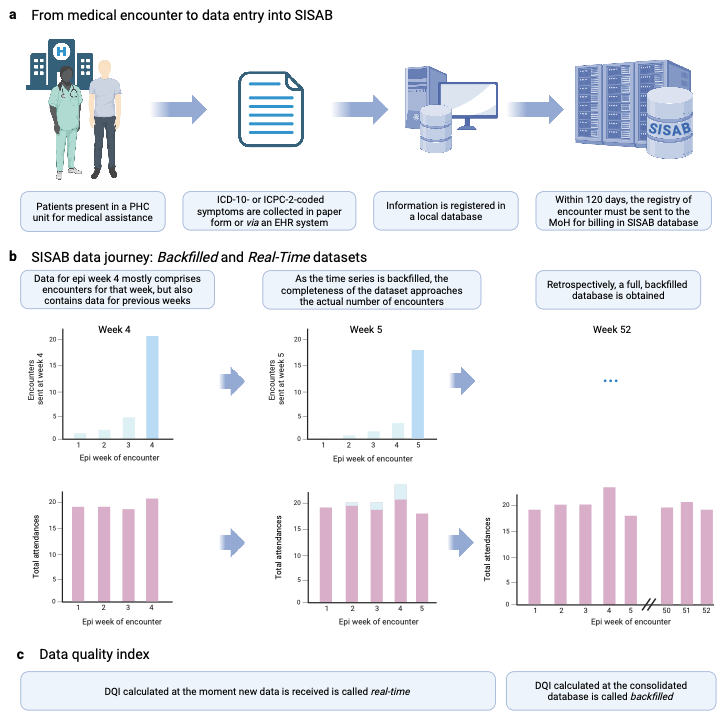


Figure S1. Schematic diagram of the methodology used in the study.

Table S1. Distribution of detected outbreak warnings according to concordance between backfilled and real-time Primary Health Care datasets, and to levels of completeness and timeliness of the real-time dataset, in the study period.

|  | **Concordant**  **n (%)** | **Non-concordant**  **n (%)** |
| --- | --- | --- |
| **Overall** | 12,538 (65.0) | 6,737 (35.0) |
| **Completeness (%)** |  |  |
| 0.0 | 0 (0.0) | 70 (100.0) |
| 12.5 | 0 (0.0) | 31 (100.0) |
| 25.0 | 1 (1.2) | 81 (98.8) |
| 37.5 | 3 (2.4) | 120 (97.6) |
| 50.0 | 4 (0.6) | 672 (99.4) |
| 62.5 | 9 (1.3) | 696 (98.7) |
| 75.0 | 85 (16.2) | 441 (83.8) |
| 87.5 | 337 (26.2) | 947 (73.8) |
| 100.0 | 12,099 (76.7) | 3,679 (23.3) |
| **Timeliness*** | 99 (96-100)* | 83 (39-98)* |
| *Median (IQR) |  |  |

Table S2. Distribution of detected outbreak warnings according to concordance between backfilled and real-time Primary Health Care datasets, and to the Data Quality Index in the study period (comprising a total of 23 epidemiological weeks)

|  | **Concordant**  **n (%)** | **Non-Concordant**  **n (%)** |
| --- | --- | --- |
| **Overall** | 12,538 (65.0) | 6,737 (35.0) |
| **Suitable DQI** | 11,656 (80.4) | 2,845 (19.6) |
| **Unsuitable DQI** | 882 (18.5) | 3,892 (81.5) |
| **Weeks with suitable DQI (%)** |  |  |
| ≤20 | 276 (8.1) | 3,114 (91.9) |
| 20-40 | 259 (34.8) | 486 (65.2) |
| 40-60 | 462 (48.9) | 482 (51.1) |
| 60-80 | 2,276 (78.4) | 628 (21.6) |
| 80-100 | 9,265 (82.0) | 2,027 (18.0) |
