# Supplementary material for: Impact of Primary Health Care Data Quality on Infectious Disease Surveillance in Brazil: Case Study
Source: JMIR Public Health Surveill. 2025 Feb 21;11:e67050. doi: 10.2196/67050 (PMC11870279; doi:10.2196/67050)
Supplement: Multimedia Appendix 2 [file publichealth-v11-e67050-s002.pdf]

please check for readability and clarity:
we found that high levels of completeness (100%, which means that every data point in the 8-week rolling window should be filled with any PHC encounter register) yield the best results

The sentence is clear, but it can be slightly refined for better readability:

"We found that achieving high levels of completeness (100%, meaning that every week in the 8-week rolling window contains at least one PHC encounter record) yields the best results."

please check for readability "Our findings revealed that municipalities with more than 60% of weeks featuring a suitable DQI demonstrated the highest proportion of concordant warnings between historical and real-time datasets"

Your sentence is clear and readable. However, a slight rephrasing can improve the flow and clarity:

"Our findings revealed that municipalities with over 60% of weeks featuring a suitable DQI had the highest proportion of concordant warnings between the historical and real-time datasets."

check for clarity and possible mistakes "There were 198,335,762 registers of encounters in the historical PHC dataset, corresponding to an average of 8,623,294 encounters per week between October, 10, 2023 and March, 10, 2024. Using the historical dataset, the EWS detected a median (IQR) of 4 (2-5) outbreaks per municipality. "

Your text is mostly clear, but a few adjustments can enhance clarity and readability:

"There were 198,335,762 recorded encounters in the historical PHC dataset, corresponding to an average of 8,623,294 encounters per week between October 10, 2023, and March 10, 2024. Using the historical dataset, the EWS detected a median (IQR) of 4 (2-5) outbreaks per municipality."

Changes made:

1. Changed "registers of encounters" to "recorded encounters" for clarity.
2. Removed the comma after "October" and "March" in the date range for correctness.
3. Added a comma after "2024" to improve readability.

in the context of a technical report on evaluating the data quality of a database, check the following sentence for clarity, concision, precision and check for possible mistakes: "We evaluated the timeliness dimension of SISAB by counting the number of weeks between the date of the PHC encounter and the date the data was registered in the system. "

The sentence is clear and concise overall, but it could be improved for precision and readability. Here's a revised version:

"We assessed the timeliness of SISAB data by calculating the number of weeks between the primary healthcare (PHC) encounter date and the date the data was entered into the system."

This revision simplifies the sentence and clarifies what is being evaluated and how.
